# Biodegradability Evaluation of Polymers by ISO 14855-2

**DOI:** 10.3390/ijms10083635

**Published:** 2009-08-18

**Authors:** Masahiro Funabashi, Fumi Ninomiya, Masao Kunioka

**Affiliations:** National Institute of Advanced Industrial Science and Technology (AIST) / 1-1-1 Higashi, Tsukuba, Ibaraki 305-8565, Japan; E-Mails: f.ninomiya@aist.go.jp (F.N.); m.kunioka@aist.go.jp (M.K.)

**Keywords:** biodegradability, polycaprolactone, poly(lactic acid), compost, evaluation, ISO 14855-2, ISO/DIS 10210

## Abstract

Biodegradabilities of polymers and their composites in a controlled compost were described. Polycaprolactone (PCL) and poly(lactic acid) (PLA) were employed as biodegradable polymers. Biodegradabilities of PCL and PLA samples in a controlled compost were measured using a Microbial Oxidative Degradation Analyzer (MODA) according to ISO 14855-2. Sample preparation method for biodegradation test according to ISO/DIS 10210 was also described. Effects of sizes and shapes of samples on biodegradability were studied. Reproducibility of biodegradation test of ISO 14855-2 by MODA was confirmed. Validity of sample preparation method for polymer pellets, polymer film, and polymer products of ISO/DIS 10210 for ISO 14855-2 was confirmed.

## 1. Introduction

Polymers are widely used in various industrial fields due to their merits, such as light weight, resistance to chemicals, resistance to the environment, easy processing, etc. It is difficult for polymers to be treated after use due to their resistance to the environment. When polymers are disposed of in a natural environment, they remain for a long time without degradation. One of the solutions to this problem of polymers after use is the development of biodegradable polymers. Biodegradable polymers can be biodegraded eventually by microorganisms in the natural environment into carbon dioxide (CO_2_) and water (H_2_O). Various types of biodegradable plastics have been developed. Among these biodegradable plastics, polycaprolactone (PCL) was developed as a biodegradable plastic of the aliphatic polyester type derived from the chemical synthesis of crude petroleum. PCL has good water, oil and chlorine resistance. It has a low melting point (ca. 60 °C), low melt viscosity and it is easy to process. The low melting-point of PCL is expected to be suitable for composting as a means of disposal, since the temperature obtained during composting routinely exceeds 60 °C. PCL is mainly used in thermoplastic polyurethanes, resins for surface coatings, adhesives and synthetic leather and fabrics. It also serves to make stiffeners for shoes and orthopedic splints, and fully biodegradable compostable bags, sutures, and fibers. PCL is often mixed with starch to obtain a good biodegradable material in order to reduce its price. Poly(lactic acid) (PLA) products have become widely used because of their biodegradability, hydrodegradability and the biorenewable profile with its source as vegetables such as corn. This allows both the prevention of environmental pollution and the use of renewable resources for a sustainable society. Biorenewable materials are materials made from biomass with absorbed carbon dioxide from the global atmosphere in modern times. The use of biorenewable materials causes a decrease in oil consumption, and the production of the CO_2_ in the atmosphere from the incineration of such wastes is the same as the CO_2_ fixed during photosynthesis by plants from the atmosphere nowadays. This concept is called “zero-emission”. In this way, global warming will be reduced. Considering these points, the use of biodegradable and biorenewable materials is expected to be promoted. For increased use of these materials in actual commercial markets, international standards for the evaluation of their biodegradability are very important. Consumers require that products made of biodegradable plastics should display their biodegradability according to international standards.

**Figure 1.**
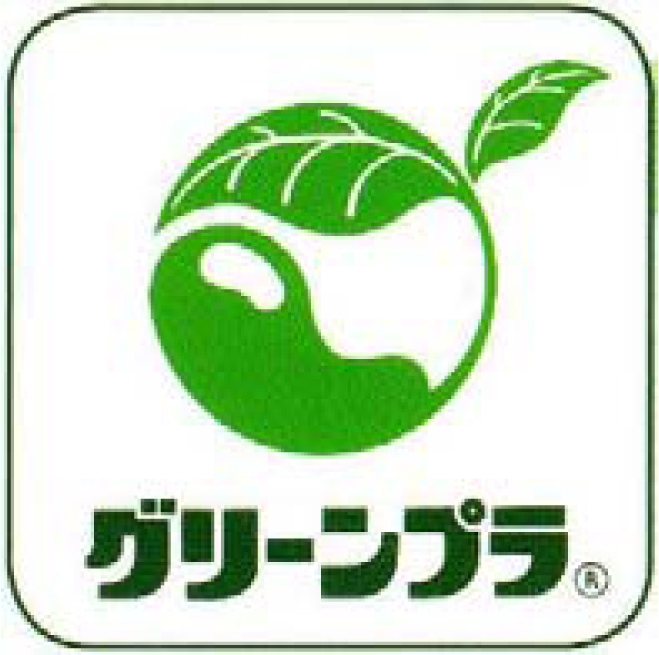
“GreenPla” mark for GreenPla Certification System by JBPA.

**Figure 2.**
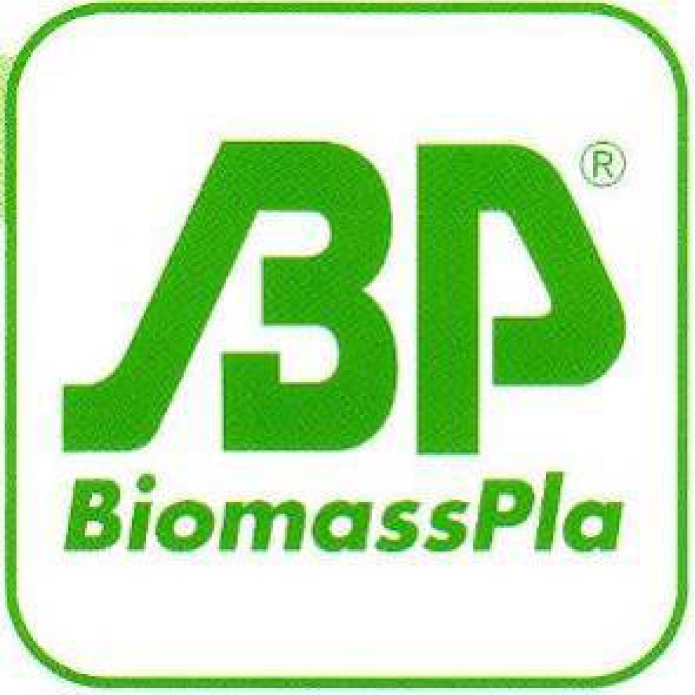
“BiomassPla” mark for BiomassPla Certification System by JBPA.

The Japan Bioplastics Association (JBPA) is a organization voluntarily founded by the major Japanese plastics producing companies and others focused on plastics, which was established with the aim of promoting the popularization of biodegradable plastics and biomass-based plastics, which play an important role in solving the recycling-technical problems [1]. JBPA has established two identification systems related to biodegradable plastics products and biomass-based plastics products. That is, the GreenPla Identification System and the BiomassPla Identification System. There are logo marks for these systems as shown in Figures 1 and 2.

The International Organization for Standardization (ISO) is a worldwide federation of national standard bodies (ISO member bodies) [2,3]. The work preparing International Standards is normally carried out through the ISO Technical Committee (TC). Each TC has subcommittees (SC) for related topics and the SC has several working groups (WG). TC61 is a TC concerning “Plastics”. WG22 was created as a new WG for “Biodegradability” under SC5 on “Physical chemical properties” by TC61 in 1993. A convener of WG22 was Dr. Sawada with the support by JBPA, since Japan insisted that it was necessary for a new working group on “biodegradability” to be established in TC61. Standards for the biodegradability of plastics in the ISO are discussed under TC61/SC5/WG22. Recently, a discussion about the concept of the bio-based content of plastics has been proposed. The ISO standards list about the biodegradation of plastics discussed under TC61/SC5/WG22 is shown as Table 1. Eleven ISO standards, including one standard under discussion are maintained under TC61/SC5/WG22. There are nine standards for the biodegradation testing of plastics. One standard for the specification and one standard for sample preparation for the biodegradation tests exist. Nine standards for the biodegradation test can be classified by the test environment such as an aqueous solution, a compost, and soil, as shown in Table 1. ISO 14855-1 and ISO 14855-2 are biodegradation tests in a compost, ISO 14855-1 is a pilot-scale test and ISO 14855-2 is a laboratory-scale test. According to ISO 14855-1, 50 g of a sample is required for one measurement and three test samples are required for the one measurement. Therefore, at least 150 g of a sample is required for one measurement by ISO 14855-1. On the other hand, 10 g of a sample is required according to ISO 14855-2. The Biodegradable Plastics Society (BPS), which was the former JBPA society, planned a round-robin test of biodegradation test based on ISO 14855-2 before being established as an ISO standard. After the round-robin test in Japan for four years, Dr. Uematsu with support by BPS (JBPA) proposed ISO 14855-2 as a new biodegradation evaluation method in a laboratory scale on TC61/SC5/WG22 in 2003. An international round-robin test was proposed in order to confirm this method and then seven countries (Sweden, Italy, Belgium, China, India, USA and Japan) have researched the ultimate aerobic biodegradability of plastic materials under the same conditions using the Microbial Oxidative Degradation Analyzer (MODA) apparatus based on the round-robin test (2004–2006) [4]. Our laboratory joined this round-robin test as the representative of Japan. Samples for the round-robin test were prepared in our laboratory. It is known that the biodegradabilities of sample depend on the sample’s size and shape. The biodegradation of various samples based on ISO 14855-2 have been studied using the international round-robin test up to now [4–10]. From these results, a sample preparation method has also been investigated [6–8]. This sample preparation method was arranged for all the biodegradation tests in the ISO standard and was proposed as a new ISO standard for TC61/SC5/WG22 by Dr. Kunioka. This standard is ISO/DIS 10210, now under discussion as shown in Table 1. There are few articles concerning the biodegradability of polymers in a controlled compost. Especially, the studies of the biodegradability of polymers measured by ISO 14855-2 are very rare. The biodegradation of polymer composites consisting of PLA and cotton fibers has been studied [5]. The biodegradation of poly(butylene succinate) (PBS) has been reported [10].

In this review, the biodegradation of various types and shapes of biodegradable polymers and their products were investigated in order to confirm ISO 14855-2 and ISO/DIS 10210. The biodegradabilities of PCL and PLA in controlled compost, measured on the basis of ISO 14855-2 by MODA, are described. The sample preparation method for this measurement according to ISO/DIS 10210 was also described. The biodegradabilities of various types of samples, such as powders, plates, and films, were measured by MODA. Additionally, the biodegradation of PLA composites with cotton fibers in a controlled compost was investigated.

## 2. Experimental Section

### 2.1. Materials

Polycaprolactone (PCL) and poly(lactic acid) (PLA) were used as biodegradable plastics for the biodegradation tests. The commercial PCL pellets were kindly supplied by Daicel Chemical Industries, Ltd., Japan. The commercial PLA pellets were kindly supplied by Mitsui Chemicals, Japan. The commercial PLA film with a 25 μm thickness was kindly supplied by Unitika, Ltd., Japan. Returnable cups made of PLA which were actually used at the Aichi EXPO 2005 in Japan were used as an example of products made of biodegradable plastics. PLA and PLA composites were prepared in our laboratory by the polymerization of l-Lactide (LA, (3*S*, 6*S*)-*cis*-3,6-dimethyl-1,4-dioxane-2,5-dione, Tokyo Kasei, Japan) and aluminum triflate (trifluoromethanesulfonate, Al(OTf)_3_; Aldrich) with and without absorbent cotton (Iwatsuki Co., Japan) [5].

The cellulose powder of thin-layer chromatography grade with a particle size of less than 20 μm (cellulose microcrystalline; Merck, Germany), soda talc (sodium hydroxide on support, granulated about 1.6–3 mm; Merck, Germany), soda lime (sodium hydroxide on support, small granules about 1.5–3 mm; Kanto Chemical, Japan) and sea sand (sand washed, 425–850 μm; Wako Pure Chemical, Japan) were used for the biodegradation test by the Microbial Oxidative Degradation Analyzer (MODA) (Hissan Trade Co., Ltd., Japan) as received.

### 2.2. Preparation of Samples for Biodegradation Test

For the biodegradation test in the compost by MODA, samples were made based on their shapes such as powders and plates. The powder type samples were prepared by mechanical crushing. The polymer pellets, returnable cup, and films were crushed into powders. Samples with dry ice were mechanically crushed by a rotating mixer with titanium blades. The crushing was carried out 15 times for 3 min each at 5 min intervals to prevent overheating of the motor in the mixer. After drying under reduced pressure at room temperature, the sample powder was separated using sieves of 30 mesh (500 μm), 60 mesh (250 μm), and 125 mesh (125 μm). Standard sieves with a guarantee were used. The powders were separated by size. That is, 0–125 μm, 125–250 μm, 250–500 μm, etc. The 125–250 μm powders are recommended for the biodegradation test of the ISO standards in ISO/DIS 10210. The sieves with crude polymer powders were placed on a sieve vibrator and vibrated for 15 min.

The actual product samples were cut into plates for the biodegradation tests by MODA. The PLA returnable cup was cut into 1 cm square plates using the nippers. The PLA powders from cups were obtained from PLA plates by a rotating mechanical mixer. The PLA films were cut into 1 cm and 5 cm squares. Crushed PLA films were prepared by a mechanical mixer. The composite samples were cut into blocks for the biodegradation tests by MODA. The maximum length of each block was less than 5 mm.

### 2.3. Preparation of Controlled Compost

The controlled compost (YK-5, Hissan Trading Co., Ltd., Japan) for MODA based on ISO 14855-2 was prepared as follows. The waste material of used wood blocks for growing mushrooms and also chicken droppings were composted for 7 months. During this period, mature compost was prepared. After preparation, this obtained compost was sieved using a 4.7 mesh (4 mm) sieve, kept at room temperature and prevented from drying. The properties of this compost are shown in Table 2. This compost can be kept at room temperature for a long time, at least five years. Before using this compost (YK-5), an activation step (preincubation) was required to recover the biological activities for the respiration and biodegradation by microorganisms.

The compost (ca. 80 g) was mixed sea sand (425–850 μm, ca. 320 g). The controlled compost was prepared by adding adequate water to this mixture in order to control water content of the original compost over 80%, where the water content is determined as a weight ratio of total water (adding water and water included the compost) to volatile solids of the original compost. The weight indicated in parenthesis is that for one reaction vessel. Preincubation was performed once for the total amount of blanks and samples using a big container (5 L). Sea sand was added to make good homogeneous conditions and a better aerobic environment inside the compost. This compost for the activation step was mixed once a day and the water content of 65 wt% for 7 days at 58 °C was controlled.

### 2.4. Biodegradation Measurement Based on ISO 14855-2

**Figure 3.**
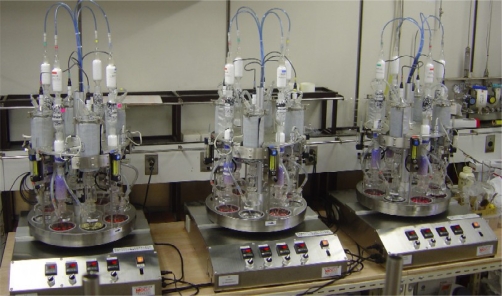
Outside of the Microbial Oxidative Degradation Analyzer (MODA).

**Figure 4.**
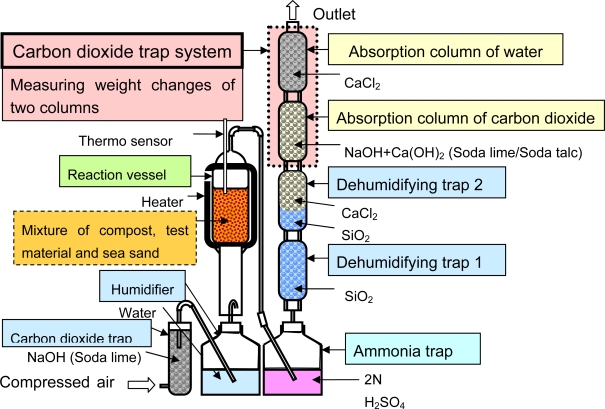
Scheme of the Microbial Oxidative Degradation Analyzer (MODA).

A biodegradation test was performed using the MODA apparatus in controlled compost at 58 °C as shown in Figure 3. The scheme is shown in Figure 4. A 10 g sample was well mixed in the activated compost with sea sand (ca. 400 g) and transferred to a reaction vessel. Compost with no sample was used as the blank to determine the respiration activity of this compost. The biodegradation tests were performed at 58 °C at a 10 mL/min air (CO_2_-free) flow rate for 30–130 days. The activated compost used in this study produced 50 mg CO_2_ per gram of volatile solids of this compost over the first 10 days. In almost all cases, the number of experimental replicates of the blank or sample was two (duplicate). The produced CO_2_ amounts were measured once a day by measuring the weights of an absorption column for carbon dioxide and an absorption column for water. The degree of biodegradation percentage was calculated from the produced CO_2_ amount from which was subtracted the respiration CO_2_ amount determined from a blank, and the theoretically produced CO_2_ amount of the added sample. For example, 10 g of PLA could be changed with 18.3 g of CO_2_ which was the theoretical amount for the 100% biodegradation. Once a week, the sample and compost were well mixed and the water content was controlled. The absorbed CO_2_ amounts for the absorption columns reached 40% of the theoretical absorption capacity, and the chemicals (soda lime and soda talc) inside the columns were changed. This test method is based on ISO 14855-2.

### 2.5. Particle Size Measurement of Powder Samples

The particle sizes of the powder samples were measured by optical microscopy using a digital camera. The diameter lengths of at least 100 particles were measured from optical microscopy photographs for one fractionated polymer powder. The maximum length of each particle was measured as the diameter of each particle. The average diameter of the polymer powders was determined to be the number average of these diameters in the photographs.

## 3. Results and Discussion

### 3.1. Shapes of Reference Materials for Biodegradation Tests

**Figure 5.**
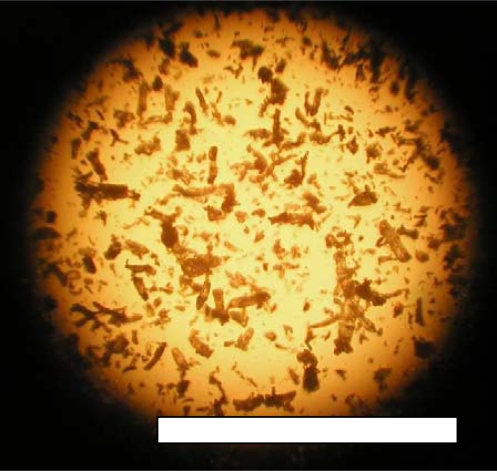
Cellulose powders as reference material; bar 0.5 mm.

Cellulose powders of thin-layer chromatography grade with a particle size of less than 20 μm were used. These cellulose powders were employed as a reference material for the biodegradation test in the compost using the Microbial Oxidative Degradation Analyzer (MODA). This is clearly indicated in ISO 14855-2. A photograph of these cellulose powders are shown in Figure 5. The shapes of the cellulose powders are angular.

### 3.2. Shapes of Powder Samples for Biodegradation Tests

**Figure 6.**
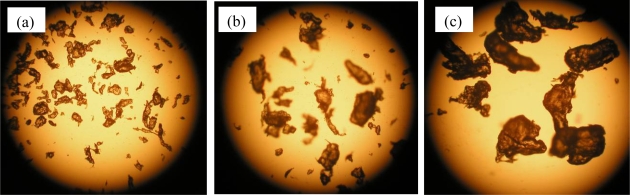
Crushed PCL powders; bar 0.5 mm [7]. (a) 0–125 μm. (b) 125–250 μm. (c) 250–500 μm.

**Figure 7.**
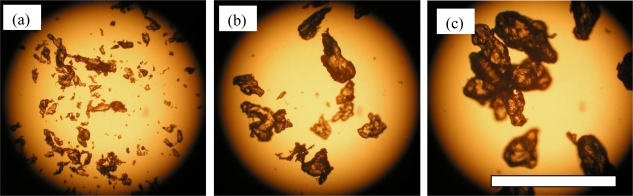
Crushed PLA powders; bar 0.5 mm [6]. (a) 0–125 μm. (b) 125–250 μm. (c) 250–500 μm.

**Figure 8.**
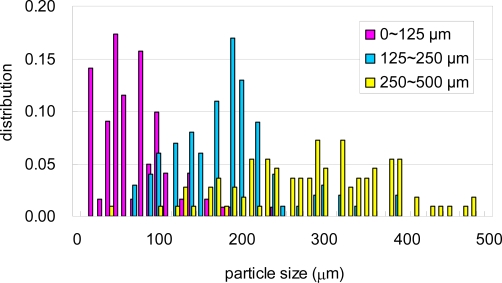
Size distributions of crushed PCL powders separated by sieves of 125 μm, 250 μm, and 500 μm [7].

**Figure 9.**
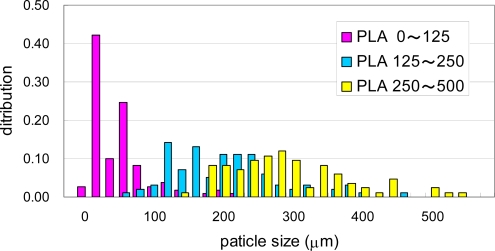
Size distributions of crushed PLA powders separated by sieves of 125 μm, 250 μm, and 500 μm [6].

The PCL pellets and PLA pellets were mechanically crushed into PCL powders and PLA powders as described in the experimental section [6,7]. These powders were separated by sieves with 125 μm, 250 μm and 500 μm square holes. Photographs of the separated PCL powders are shown in Figure 6. The PCL powders of 0–125 μm in (a), 125–250 μm in (b), and 250–500 μm in (C). The PLA powders of 0–125 μm in Figure 7 (a), 125–250 μm in Figure 7 (b), and 250–500 μm in Figure 7 (C), respectively. The maximum length of each particle in the photographs was measured as the diameter of the particles. The size distributions of the crushed powders of PCL and PLA are shown in Figures 8 and 9, respectively. The distributions of the powders for three ranges divided by the sieves are shown in the figures by different colors. From the photographs and distributions, it is observed that the powder can be separated by sieves. It was found that the preparation method of the powder samples in this study is effective. The fine powders of polymers could be obtained by this method. The recoveries and averaged diameters of the PCL and PLA powders for each range fractionated by the sieves are shown in Tables 3 and 4, respectively [6,7]. The powder samples with 125–250 μm sizes are recommended in ISO/DIS 10210 for the biodegradation tests of ISO standards including ISO 14855-2.

### 3.3. Shapes of Film Samples for Biodegradation Tests

**Figure 10.**
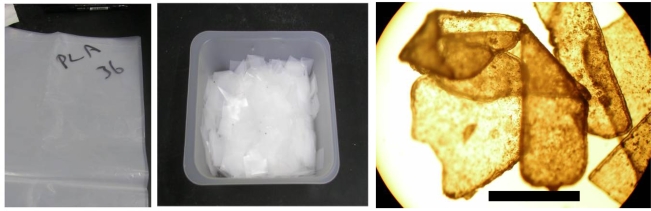
(a) PLA film from UNITIKA, Ltd. (b) PLA film cut into 1 cm square. (c) PLA films broken by a mixer through 125 μm sieve; bar=0.1 mm.

The biodegradation of the PLA films in a controlled compost was measured by the MODA apparatus. The biodegradabilities for two kinds of film samples with different shapes were investigated. That is, one was cut films with 1 cm or 5 cm squares, and the other was film crushed by a mechanical mixer. The photographs of the original films, cut films and crushed films are shown in Figure 10. For ISO/DIS 10210, film type samples are recommended as the shapes for the biodegradation tests. The sizes of the crushed film are greater than 125 μm, since crushed films were folded during crush as shown in Figure 10 (c). It is difficult for thin film to be crushed into small specimens. The apparent density of the crushed film is much smaller than that of cut film or folded film. So, cut films of 1cm square are recommended as samples for the biodegradation tests of ISO standards.

### 3.4. Shapes of PLA Returnable Cup for Biodegradation Tests

**Figure 11.**
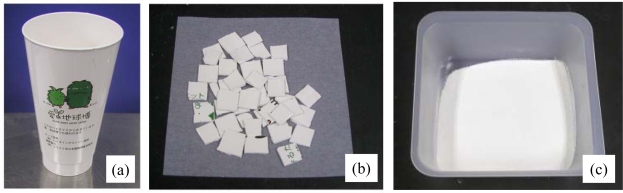
(a) PLA returnable cup. (b) PLA plates cut of 1 cm square from cup. (c) PLA powders crushed from plates [8].

**Figure 12.**
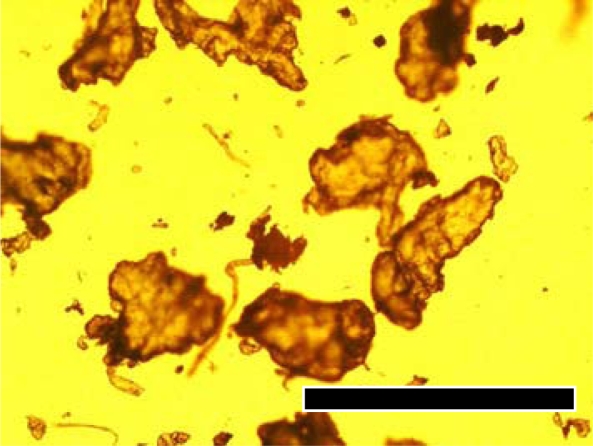
PLA powders crushed from returnable cup fractionated by sieves of 125 μm and 250 μm; bar=0.5 mm [8].

**Figure 13.**
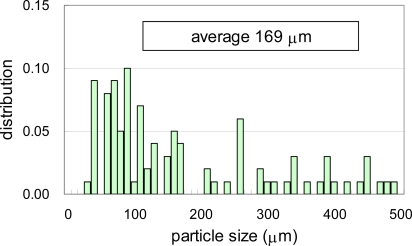
Distribution of PLA powders crushed from returnable cup collected between sieves of 125 μm and 250 μm [8].

The biodegradability of the returnable cup made of PLA for Aichi EXPO 2005 was measured using cut plates or crushed powder from the cup by the MODA apparatus. The plate specimens were 1 cm squares cut by a nipper. The powders were mechanically crushed from plates using a mixer by the same procedure as that for pellets. Photographs of the original cup, cut plates and crushed powder are shown in Figure 11. The powders were separated by a sieve with 250 μm holes. A microphotograph of powder cut off greater size than 250 mm is shown in Figure 12. The sizes of the powders in the photographs were measured. The distribution and average size are shown in Figure 13. The fine powders of the cup could be obtained by this method.

### 3.5. Shapes of PLA Composite Sample with Cotton Fibers for Biodegradation Tests

**Figure 14.**
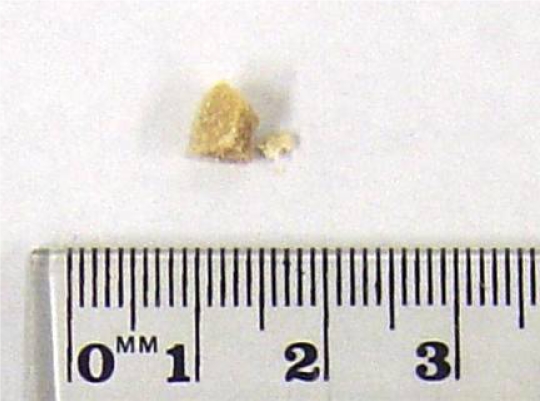
PLA composite specimen cut from column shaped sample [5].

The biodegradability of the composite samples of PLA and cotton fibers was measured according to ISO 14855-2 [5]. Composite samples with a column shape were prepared by direct molding from lactide, triflate and cotton fiber [5]. The composite samples were cut into small specimens for the biodegradation test in the controlled compost by MODA. One of cut specimens is shown in Figure 14. Sizes of these cut specimens are less than 5 mm as shown in Figure 14. For the actual products, blocks from the samples are recommended for use by the biodegradation tests in ISO/DIS 10210.

### 3.6. Biodegradation Test Results of Reference Materials in Controlled Compost by ISO 14855-2

**Figure 15.**
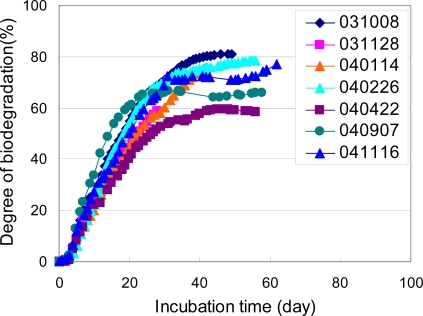
Biodegradabilities of cellulose powders as reference materials in controlled compost at 58 °C [6–8].

The biodegradation tests in a controlled compost based on ISO 14855-2 were performed using the MODA apparatus. Results of the biodegradation tests are shown in Figure 15. According to ISO 14855-2, the cellulose powders are used as reference materials. The same compost was employed for the biodegradation test by MODA as described in this paper. The preincubation procedure was also the same as for all the tests. The numerical digits in the figure indicate starting date of the biodegradation tests. Although some scatter is observed, all the biodegradation curves are almost the same. These results suggest that the cellulose powders are biodegraded by almost the same process. That is, cellulose powders can be biodegraded at least 60% after 40 days. The biodegradability of cellulose in the same compost is almost the same for different times. The reproducibility and repeatability of the biodegradation test for the cellulose powders agreed well. These results suggest that the cellulose powders are effective as a reference material for ISO 14855-2. No induction period of biodegradation curves is observed. It is thought that there is no induction period in biodegradation curve, since cellulose can be directly degraded by enzymes such as lipase and cellulase. The biodegradation tests of cellulose powders were terminated when the biodegradation curve show plateau, since that the termination of the test is regulated in ISO 14855-2. Biodegradation of samples based on ISO 14855-2 is evaluated by evolved carbon dioxide, since the carbon atoms from samples left in a compost can not be accurately evaluated. Therefore, final degrees of biodegradation of cellulose are from 60 to 80% for biodegradation tests of cellulose by ISO 14855-2 and do not equal to 100%. 60 to 80% of carbon atoms from samples become carbon dioxide by microorganism and left 20 to 40% of carbon atoms change the others such as body of microorganisms.

### 3.7. Biodegradation Test Results of PCL Powders in Controlled Compost by ISO 14855-2

**Figure 16.**
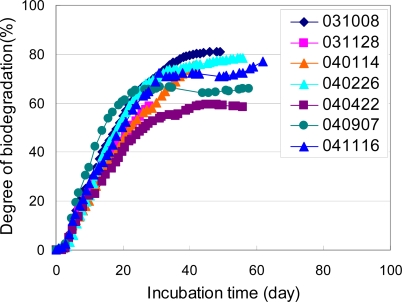
Biodegradabilities of PCL powders in controlled compost at 58 °C [7].

**Figure 17.**
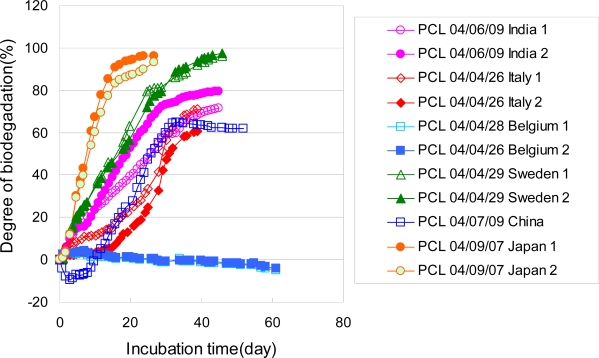
Biodegradabilities of PCL powders of 125–250 μm in controlled compost at 58 °C by round-robin test [4].

The biodegradation of PCL powders in a controlled compost were measured based on ISO 14855-2 [7]. The PCL powders were prepared by mechanically crushing and sieving. Sizes between 125 and 250 μm were used for the biodegradation test. The degrees of biodegradation of these PCL powders based on the ISO 14855-2 method using the MODA instrument measured at different times are shown in Figure 16. The numbers indicated in the figure show the starting date of the biodegradation tests. All the degrees of biodegradation of the PCL powders at different times show almost the same curves. The reproducibility of the biodegradability of the PCL powders in this method using the MODA instrument is good. It is thought that these PCL powders are suitable for the biodegradable testing method as the reference material. No induction time was observed for the biodegradation of the PCL powders, although the induction times of around 10 days were observed for the biodegradation of the PLA powders by this method [6]. Except for one result indicated as “114”, all the PCL powders are biodegraded over 60% after 20 days. The PCL powders prepared by crushing and sieving according to ISO/DIS 10210 were used as the samples of the international round-robin test for ISO 14855-2 in ISO/TC61/SC5/WG22 [4]. The results of the international round-robin test are shown in Figure 17. It was found that the PCL powders can be biodegraded over 60% for 30 days except for the results from Belgium. The different compost derived in each country mainly causes difference among the biodegradation curves. There is no induction period in biodegradation curves of PCL, since PCL can be also degraded by enzymes of lipase and cellulase.

### 3.8. Biodegradation Test Results of PLA Powders in Controlled Compost by ISO 14855-2

**Figure 18.**
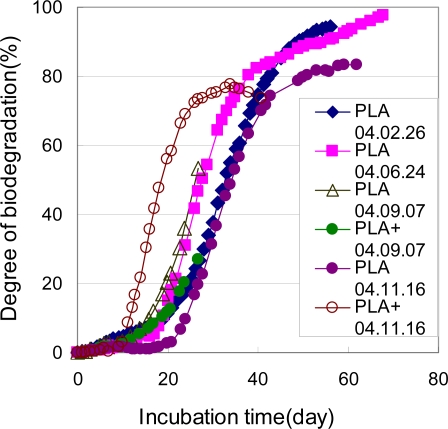
Biodegradabilities of PLA powders of 125–250 μm in controlled compost at 58 °C [6].

The biodegradation of the PLA powders in a controlled compost was measured by MODA. The PLA powders were prepared by the same procedure as that for the PCL powders. Figure 18 shows the biodegradation of the PLA measured at four different times by the MODA instrument in a controlled compost at 58 °C. After the induction periods about 10–20 days, the degradation rates were accelerated. After about 50 days of incubation at 58 °C, the degree of biodegradation reached 80%. It was postulated that the molecular weight of the PLA decreased by hydrolysis during the period of induction. If the molecular weight of the PLA reached the value (PLA oligomer) which can be metabolized by microorganisms in the compost, the biodegradation of PLA was actively done and much CO_2_ was evolved. For these PLA powders, this reproducibility of the biodegradation evaluation was found to be good during these seven different time measurements. This reproducibility is thought to be acceptable for the ISO 14855-2 method. The effect of the powder sizes on the biodegradabilities of PLA powders was investigated [6]. For the PLA with a size distribution between 0 and 125 μm, the biodegradation speed was two times faster than that between 125 and 250 mm. An over 100% degradation was observed in this sample. This may be due to the more active respiration of the microorganisms in the compost by the well metabolized carbon source, *i.e.,* the fine PLA powders. This fact suggests that powders less than 125 μm in size should not be used for the biodegradation tests such as ISO 14855-2. This elimination of fine particles has been proposed in ISO/DIS 10210.

### 3.9. Biodegradation Test Results of PLA Returnable Cup in Controlled Compost by ISO 14855-2

**Figure 19.**
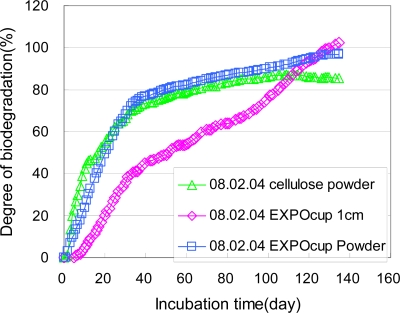
Biodegradation test of cellulose powders, PLA powders and PLA plates from PLA cup by ISO 14855-2 method using MODA in controlled compost at 58 °C.

The biodegradabilities of the PLA returnable cup, which was used in Aichi EXPO 2005 were measured according to ISO 14855-2 [8]. The biodegradation tests of cellulose powders, PLA plates and PLA powders based on ISO 14855-2 were performed in our laboratory using the MODA instrument in a controlled compost at 58 °C. These test results are shown in Figure 19. The PLA powders from pellets show almost the same biodegradation curve as that of the cellulose powders. These two samples, the PLA powders and cellulose powders, gradually degraded for the first three days. The degrees of biodegradation of these samples monotonously increased with the increasing incubation time when the incubation time reached 30 days. The degrees of biodegradation of these samples maintained ca. 80% at incubation times greater than 30 days. For the plates from the PLA cup, the biodegradation speed is slower than those of powders from the cup and cellulose powders. The degree of biodegradation of the plate samples was almost 0 for the first 15 days. The degree of biodegradation of the plates monotonously increased with the increasing incubation time. The degree of biodegradation of the plates maintained ca. 80% when the incubation time reached 100 days. The effect of the sample shapes on the biodegradation speed can be clearly observed, although the final biodegradability’s of the PLA powders and plates from the PLA cup and cellulose powders are the same, *i.e.,* 80% [6]. The testing conditions, such as the kind of compost, preincubation, and temperature, for biodegradation tests in Figure 19 is the same as those in Figure 18. The induction period is different, although the biodegradation gradients in Figures 18 and 19 are almost the same. Some additives, such as plasticizer and compatilizer, are usually used for actual products, although the components of the PLA cup are unknown. It is thought that these additives may affect the biodegradation process of PLA and reduce the induction period. The biodegradation data of the PLA cup and PLA pellets can be precisely compared with ease to others measuring the biodegradation of regulate sized powders of the PLA cup and PLA pellets. It is necessary to regulated sample shapes when the consumers want to precisely compare the biodegradability data of the actual products.

### 3.10. Biodegradation Test Results of PLA Films in Controlled Compost by ISO 14855-2

**Figure 20.**
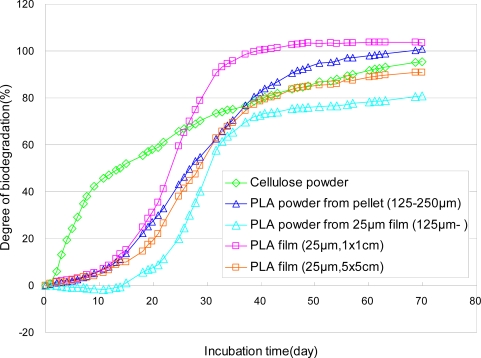
Biodegradabilities of PLA powders (125–250 μm), crushed PLA films (collection by 125 μm sieve, 25 μm thickness), PLA films (1 cm × 1 cm × 25 μm), and PLA film (5 cm × 5 cm × 25 μm) in controlled compost at 58 °C by MODA.

The biodegradation tests of the PLA films were performed by MODA according to ISO 14855-2. Films were cut into 1 cm squares and 5 cm squares by a cutter. The crushed films were obtained from films using a mechanical mixer and elimination of the small pieces less than 125 μm. The PLA powders between 125 and 250 μm obtained from the pellets by a mechanical mixer and cellulose powders were employed for comparison. Results of the biodegradation tests of the cellulose powders, PLA powders from pellets, PLA films with 1 cm squares and 5 cm squares, and PLA film crushed by a mixer in a controlled compost at 58 °C are shown in Figure 20. The thickness of the film is 25 μm. The biodegradation curves of the PLA powder from the pellets and PLA 5 cm square film are almost the same. The biodegradation of the crushed PLA films is slower than those of other samples. One of the reasons is that the crushed film folded as shown in Figure 10 (c). The biodegradation of PLA 1 cm square films is faster than those of the PLA powders from the pellets and PLA 5 cm square films. For the film or sheet sample, the 1 cm square shape is regulated in ISO/DIS 10210. It is thought that this regulation is reasonable. From the results in Figure 20, the 5 cm square sample may be acceptable for the biodegradation tests.

### 3.11. Biodegradation Test Results of PLA Composites in Controlled Compost by ISO 14855-2

**Figure 21.**
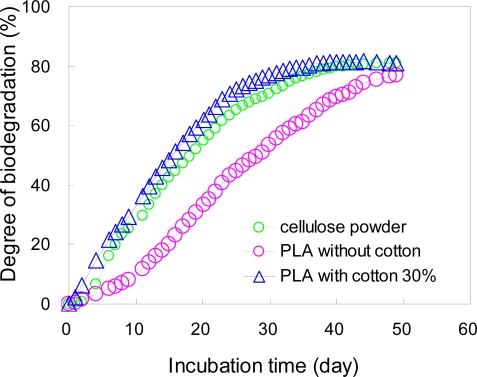
Biodegradabilities of cellulose powders, blocks of PLA, and blocks of PLA composite with 30% cotton fibers in controlled compost at 58 °C by MODA.

The biodegradation of PLA composites filled with cotton fibers in a controlled compost at 58 °C was measured by MODA according to ISO 14855-2 [5]. The results of the biodegradability of the samples with and without cotton fibers measured by MODA are shown in Figure 21. The results of the blank sample, the L0 and sample filled with 30 wt% cotton fibers, the LC30, are shown in the figure. The biodegradability of the LC30 sample is higher than that of the L0 sample during an initial stage of 10 days. This result seems to be related to the absorbed water by the fibers in the samples [5]. For the sample having a high water absorbance, evolved carbon dioxide during the sample biodegradation can be directly measured using MODA. The difference between the L0 and LC30 is clearly observed in Figure 21. PLA without cotton shows the induction period, which is observed PLA powders in Figure 18. For composite samples, cotton fibers were initially biodegraded by enzyme, such as lipase and cellulase. After cotton fibers were biodegraded, left PLA was porous. This porous structure accelerated the water absorbance and this PLA could biodegrade faster than PLA without cotton. After 50 days, the biodegradability of both samples reached 80% in the compost at 58 °C. MODA is a better method than the weight change measurement in the compost for the biodegradation analysis, especially for a sample including absorbed water such as these PLA composites. The biodegradation test based on ISO 14855-2 using MODA is applicable for not only polymer samples, but also composite samples.

## 4. Conclusions

The biodegradabilities of polycaprolactone (PCL) samples and poly(lactic acid) PLA samples in a controlled compost at 58 °C were investigated according to ISO 14855-2. Sample preparation of the polymer pellets, polymer films, polymer products, and polymer composites regulated by ISO/DIS 10210 were investigated for ISO 14855-2. Polymer powder samples were obtained from PCL pellets and PLA pellets by mechanically crushing and sieving. Cut and crushed film samples were obtained from the PLA films. Plate samples and powder samples were obtained from the PLA returnable cups. Block samples were obtained from the PLA composites with cotton fibers.

The biodegradations of the above samples were measured by the Microbial Oxidative Degradation Analyzer (MODA). The reproducibility and repeatability of the biodegradation tests using MODA were confirmed for cellulose powders as the reference material, PCL powders and PLA powders. Results of the biodegradation tests for the PLA films, PLA cup and PLA composites are shown.

It was found that biodegradabilities of polymer pellets, polymer films, polymer products and polymer composites in a controlled compost can be evaluated according to ISO 14855-2 and ISO/DIS 10210 using the MODA apparatus. Final degrees of biodegradation and biodegradation curves by biodegradation tests based on ISO 14855-2 for samples prepared from biodegradable polymers with various shapes based on ISO/DIS 10210 in this review show almost the same tendency. This fact indicates that samples derived from the polymers and their products based on ISO/DIS 10210 can be evaluated based on ISO 14855-2.

## Figures and Tables

**Table 1.** ISO standards on biodegradation of plastics in TC61/SC5/WG22.

**ISO No.**	**Title**	**Content**
14851	Determination of the ultimate aerobic biodegradability of plastic materials in an aqueous medium—Method by measuring the oxygen demand in a closed respirometer	
		aqueous
14852	Determination of the ultimate aerobic biodegradability of plastic materials in an aqueous medium—Method by analysis of evolved carbon dioxide	

14855-1	Determination of the ultimate aerobic biodegradability of plastic materials under controlled composting conditions—Method by analysis of evolved carbon dioxide Part 1: General method	
	
14855-2	Determination of the ultimate aerobic biodegradability of plastic materials under controlled composting conditions—Method by analysis of evolved carbon dioxide Part 2: Gravimetric measurement of carbon dioxide evolved in a laboratory-scale test	compost

16929	Plastics—Determination of the degree of disintegration of plastic materials under defined composting conditions in a pilot-scale test	
	
20200	Plastics—Determination of the degree of disintegration of plastic materials under simulated composting conditions in a laboratory-scale test	disintegration

17556	Plastics—Determination of the ultimate aerobic biodegradability in soil by measuring the oxygen demand in a respirometer or the amount of carbon dioxide evolved	soil

14853	Plastics—Determination of the ultimate anaerobic biodegradation of plastic materials in an aqueous system—Method by measurement of biogas production	
		anaerobic
15985	Plastics—Determination of the ultimate anaerobic biodegradation and disintegration under high-solids anaerobic-digestion conditions—Method by analysis of released biogas	

17088	Specifications for compostable plastics	specification

DIS 10210	Plastics - Preparation of test materials for biodegradation tests	preparation

**Table 2.** Properties of controlled compost [6–8].

**Analysis**	**YK-5**
Total dry solids (%) 1)	48
Volatile solids (%) 2)	63
pH of compost solution	6.5
Total organic carbon amount (%)	13
Total nitrogen amount (%)	2
C/N ratio	6.4

^1)^ The amount of solids obtained by taking a known volume of compost and drying at about 105 °C for 10 hours.

^2)^ The amount of solids obtained by subtracting the residue of a known volume of compost after incineration at about 550 °C.

**Table 3.** PCL powders crushed from PCL pellet and fractionated using sieves [7].

**Size region (μm) 1)**	**Recovery (wt%)**	**Average diameter (μm)**
0–125	35	75.7
125–250	30	180.7
250–500	20	297.6
500–	15	–

^1)^ Fractionated using indicated-size sieve.

**Table 4.** PLA powders crushed from PLA pellet and fractionated using sieves [6].

**Size region (μm) 1)**	**Recovery (wt%)**	**Average diameter (μm)**
0–125	25	60.8
125–250	25	214.2
250–500	31	303.9
500–	19	–

^1)^ Fractionated using indicated-size sieve.
